# Qigong and Fibromyalgia circa 2017

**DOI:** 10.3390/medicines4020037

**Published:** 2017-06-06

**Authors:** Jana Sawynok, Mary E. Lynch

**Affiliations:** 1 Departments of Pharmacology, Anesthesiology and Pain Management, Dalhousie University, Halifax, NS B3H 4R2, Canada; 2 Departments of Anesthesiology, Pain Management and Perioperative Medicine, Psychiatry, Pharmacology, Dalhousie University and QEII Health Sciences Centre, Halifax, NS B3H 2Y9, Canada; mary.lynch@dal.ca

**Keywords:** qigong, pain, fibromyalgia

## Abstract

Qigong is an internal art practice with a long history in China. It is currently characterized as meditative movement (or as movement-based embodied contemplative practice), but is also considered as complementary and alternative exercise or mind–body therapy. There are now six controlled trials and nine other reports on the effects of qigong in fibromyalgia. Outcomes are related to amount of practice so it is important to consider this factor in overview analyses. If one considers the 4 trials (201 subjects) that involve diligent practice (30–45 min daily, 6–8 weeks), there are consistent benefits in pain, sleep, impact, and physical and mental function following the regimen, with benefits maintained at 4–6 months. Effect sizes are consistently in the large range. There are also reports of even more extensive practice of qigong for 1–3 years, even up to a decade, indicating marked benefits in other health areas beyond core domains for fibromyalgia. While the latter reports involve a limited number of subjects and represent a self-selected population, the marked health benefits that occur are noteworthy. Qigong merits further study as a complementary practice for those with fibromyalgia. Current treatment guidelines do not consider amount of practice, and usually make indeterminate recommendations.

## 1. Introduction

Qigong is a traditional practice with a history of over 2500 years in China, and many forms have been developed within different contexts [1]. The health benefits of qigong are currently receiving increasing attention, and in 2009, terminology that is more familiar to Western biomedicine was proposed to facilitate further exploration of this potential [2]. Core elements of qigong practice involve movements, meditative components, attention to breathing, and promotion of a state of deep relaxation and integration. Qigong, characterized as meditative movement (MM), uses a framework of movement with unique attentional features [2,3]. A further characterization is as movement-based embodied contemplative practice in which the mind–body (mind-in-body) connection is emphasized; qualities and characteristics of movement are distinguished from conventional exercise, and contemplative aspects are considered within a framework of body awareness and somatic approaches [4]. Tai Chi, as well as several other more recently developed and contemporary somatic therapeutic techniques, are also considered under these umbrella terms [2,3,4]. MM is the more established term, and will be used throughout this report. 

Fibromyalgia (FM) is a condition involving chronic pain, sleep disturbances, fatigue, and often additional co-morbidities [5]. In the 1990s, FM was recognized as a clinical entity with tender point criteria, but updated criteria proposed in 2010/2011 and 2016 emphasize the chronic and widespread nature of the pain, the lack of refreshing sleep, and the variability of additional comorbidities [5,6]. FM also has been characterized as a central sensitivity syndrome recognizing the polymodal nature of comorbidities [7] (overlapping conditions include chronic fatigue syndrome, irritable bowel syndrome, headaches, multiple chemical sensitivity, and others). Treatment of FM involves many strategies and can include forms of complementary and alternative medicine (CAM). Treatment guidelines for FM have been developed by several professional organizations, and the most recent versions of such guidelines specifically reference MM as part of CAM within a comprehensive consideration of treatment options [8]. Table 1 provides examples of dedicated terminology used to characterize qigong in recent focused reviews of treatments for FM. Further consideration of evidence for qigong in FM occurs using more general terminology, i.e., in a systematic overview of reviews of CAM [9], an umbrella systematic review of exercise [10], and a Cochrane analysis of mind–body therapies [11]. 

This review article summarizes clinical experiences with qigong in FM in randomized controlled trials (RCTs) as well as other trial forms, and considers research challenges inherent in continued exploration of the health benefit potential of this practice. 

## 2. Clinical Evidence and Observations

Clinical exploration of effects of qigong in FM to date includes RCTs (Table 2) as well as pilot studies, an observational trial, and case reports (Table 3). Both quantitative and qualitative information is available.

There are challenges to assessing effects of qigong in FM, and these have led to diverse conclusions in systematic reviews and meta-analysis, as well as other reviews (Table 1). The most homogeneous body of data is represented by N = 4 RCTs involving 201 subjects in which qigong was practiced for 30–45 min daily for 6–8 weeks and there was encouragement to continue practice to 4–6 months [19,20,21,22]. In these trials, there were consistent medium-to-large effect sizes in all domains relevant to FM (pain, sleep, impact, physical and mental function) which were manifest after 6–8 weeks of practice, and benefits were sustained at 4–6 months (Table 2) [15]. Two of these trials [19,21] used a design in which the wait-list group, used as a control, was subsequently offered qigong training and constituted a delayed intervention group; both trials indicate good reproducibility between the immediate and delayed training groups.

The relationship of amount of qigong practice to outcomes requires specific attention. Effective qigong practice involves diligence and may not be for everyone (despite good intentions), and this factor needs to be considered in analyses. The benefits of qigong in core domains of FM [21] and in chronic fatigue syndrome [31] are related to the amount of practice, providing direct support for the need to consider this factor. In the FM study that compared those who practiced per protocol (≥5 h/week) with those who practiced minimally (≤3 h/week), differences in outcomes were observed in all domains, and effect sizes in the per protocol group were uniformly large (0.95–1.67) [15]. 

There are several additional reports that further document effects of qigong practice in FM (Table 3). An extension trial involving the addition of further instruction in qigong (forms of meditation) further supports the observation that benefits are related to the amount of practice [27]. Qualitative information from an RCT (qigong for 6 months) [29], and the extension trial (qigong ≥ 12 months) [27], indicates that, in addition to positive comments on core domains that essentially recapitulate the quantitative information (pain, sleep, impact, physical and mental function), health benefits in other areas also are reported (e.g., food allergies, chemical sensitivities, asthma, sleep apnea, migraines, and blood pressure). In those who practiced qigong extensively via involvement in community-based venues (1–3 years), marked health benefits occurred in FM symptomology (pain absent or minimal; other core domains improved), and there were additional benefits (e.g., irritable bowels, food sensitivities, headaches, blood pressure, and skin) [28]. In those who practiced for even longer (5–10 years), health benefits were profound, with a complete resolution of FM symptoms as well as several comorbidities (sleep apnea, high cholesterol, allergies and intolerance to multiple foods, and irritable bowel syndrome); other issues (susceptibility to infections) were much improved, and there were even marked improvements in vision [30]. Benefits in different areas occurred over time and with different time courses. Practitioners that experienced health changes over the first months of practice continued, and even increased, practice over time with the desire to further explore the health potential of the practice. Each of the trials reported in Table 2 have limitations, including the retrospective nature of case selection and the unknown generalizability of observations. Nevertheless, the profound nature of the health benefits in cases with longstanding FM and other comorbidities, where many conventional medical treatments and complementary therapies had previously been used, are particularly interesting. If they are considered to provide “proof-of-concept” information, one must conclude that qigong merits further exploration in FM. 

There is an additional report on the effectiveness of external qigong for FM in which participants received 5–7 sessions delivered by an experienced practitioner over three weeks [23]. That study reports good outcomes in 13 individuals, two of whom showed particularly marked and persistent benefits, and over three months, considered themselves cured. External qigong presents unique additional challenges in relation to the nature of the intervention and mechanisms by which health changes occur [1]. It should be noted that some RCTs included sessions of external qigong in addition to the self-practice or internal qigong that was the intervention under investigation [19,27] and that external qigong sessions were also involved in cases of extensive practice [29].

## 3. Methodological and Interpretational Challenges of Qigong Trials

Challenges to the conduct of trials on qigong, and other MM forms, have been indicated in several general, as well as FM-specific, reviews [1,2,3,4,15]. Some challenges relate to general experimental design and include: (1) the nature of controls (wait list with treatment as usual, sham interventions such as exercise, educational/psychological support via group processes); (2) blinding (this is not possible for participants, but is for data collection and analysis); (3) small sample size (RCTs of N = 14–128) (funding for such trials is difficult to obtain); (4) description of interventions (there is often limited descriptive information within the study; reference to the form of qigong provides specifics); (5) challenges of adherence during trials and of follow-up (this can bias results towards those who do obtain benefit). These factors lead to the designation of trial quality as “low” and the strength of evidence as “weak”. Other challenges are specific to the study of qigong. These include (6) heterogeneity of types of qigong (relies on local resources; styles differ at different geographic sites); (7) diverse instruction protocols and self-practice expectations (the later varies from no home practice to daily practice of 30–45 min over 6–8 weeks and continuing to 6 months); (8) a mix of potentially active factors (exercise, meditative elements, belief and expectation, psychological support); (9) skill levels of instructors and prior experience with FM populations (only some trials are informed by pilot studies); (10) development of predictive methodology to determine who is more likely to succeed with qigong practice (e.g., using health locus of control measures). With respect to continued exploration, the introduction of contemporary terminology and identification of core domains of practice in recent years represents a major advance in this area [2,3,4]. The existence of CONSORT statement variations regarding non-pharmacological interventions provides an additional framework for enhancing reporting trial quality [32]. Overview analysis of any kind must include a consideration of the amount of practice.

In the quest to better understand and improve treatment of chronic pain, the distinction between explanatory/efficacy trials and pragmatic/effectiveness trials is being elaborated [33]. Explanatory/efficacy trials have high internal validity, use pre-determined primary outcomes, have controlled conditions, use inclusion and exclusion criteria, select for homogeneous populations, compare treatments to placebo or reference drug/treatment, and provide information on mechanisms of diseases; they are highly relevant to regulatory approval and drug development. Pragmatic/effectiveness trials exhibit different characteristics, show high external validity, examine the “entire package”, use broad inclusion criteria, embed the trial within ongoing regular care, compare to usual care or standards of care, have a primary endpoint of patient care, and may be better situated for examining complex interventions such as qigong. FM is a complex chronic pain condition with multiple comorbidities, and is a challenge for patients who experience it, as well as for health practitioners who treat them [34,35]. Pragmatic trials of qigong may be more relevant than efficacy trials for providing information relating to real-world experiences of FM. Some viewpoints acknowledge pragmatic approaches to complementary therapies for FM if risk assessment indicates a low risk of harm, and within this consideration, qigong is considered potentially beneficial [36,37].

## 4. Summary and Conclusions

This manuscript summarizes beneficial effects of qigong in FM as reported in six RCTs and a further nine reports using diverse approaches. Benefits in many domains are statistically related to amount of practice [21], and this factor provides a lens through which to view the additional reports. Individual cases involving extensive practice report remarkable outcomes, both in core domains of FM as well as in other health areas. While the latter reports are limited by small numbers, selection factors, and their retrospective nature, the benefits are striking.

The study of health benefits of qigong presents unique challenges. These include challenges of representing the practice (traditional vs. contemporary terms and constructs), finding language that makes it amenable (to medical professions, as well as to those who wish to learn the practice), determining who is likely to benefit from the practice (it requires diligence and dedication, and is not for everyone), and knowing how much is needed to provide benefit for a particular condition (pain, other symptoms, comorbidities). Trials use specific protocols, but only in some instances is the protocol based on pilot studies. Benefit is related to the amount of practice, yet few studies or overview analyses stratify outcomes for this. Cases indicate that extended practice (guided by initial experiences over the first few months) is of marked benefit in many areas, but no specifics can be provided about which symptom will respond and when. The chronic pain field is now recognizing that efficacy trials and pragmatic trials have different attributes, and that pragmatic approaches can offer useful information that is relevant to the real-world experience of FM. Standard analysis of qigong trials using prescribed approaches lead to modest conclusions about merits, while a pragmatic approach, which recognizes that it is not for everyone and that multiple trial forms can provide useful information, leads to a conclusion that qigong is worthy of further exploration for FM using multiple approaches. 

## Figures and Tables

**Table 1:** Characterization of qigong within recent reviews and overviews of treatments for fibromyalgia (FM).

Characterization of Qigong	Reference	Comments, Conclusions
(1) Meditative Movement for FM (systematic review, meta-analysis)	Langhorst et al., 2013 [12]	Did not include any 2012 qigong RCTs; effect sizes in medium range; concluded high quality trials needed
(2) Qigong for FM (systematic review, meta-analysis)	Lauche et al., 2013 [13]	Considered N = 6 RCTs of qigong for FM; concluded qigong may be useful, but recommendation regarded as weak based on trial quality
(3) Complementary and Alternative Exercise for FM (meta-analysis)	Mist et al., 2013 [14]	Noted medium-to-large effect sizes; concluded little risk in recommending as component of multimodal treatment
(4) Qigong for FM (review)	Sawynok, Lynch 2014 [15]	RCTs involving daily practice for 6–8 weeks show consistent medium-to-large effect sizes in core domains, with benefits sustained at 4–6 months; concluded qigong merits continued exploration for FM
(5) Mind–body therapies for FM (as part of rheumatic diseases) (review)	Del Rosso, Maddali 2016 [16]	Notes variable results in 4 RCTs in FM (one in children); did not cite 2 adult RCTs from 2012; concluded mind–body therapies useful for overall health in rheumatic diseases

NOTE: Between 2003 and 2012, six controlled RCTs of qigong for FM in adults were published [17,18,19,20,21,22]; these originated at different sites (USA, Sweden, Canada, Italy) and reflect different forms of qigong and different regimens of practice. Four RCTs involved daily practice of qigong for 6–8 weeks [19,20,21,22]. Some reviews and analyses include a pilot study of external qigong [23], a study in children [24], and studies where qigong was part of multi-component treatment and no daily practice was involved [17,18].

**Table 2:** Summary of randomized controlled trials (RCTs) of qigong for fibromyalgia in adults.

Study Characteristics	Outcomes, Features
(1) Astin et al., 2003 [17] N = 128; N = 64 qigong, N = 64 education support Means: age 47.7 yrs, FM duration 5.0 yrs Intervention: Qigong (Dance of Phoenix) + mindfulness meditation, 2.5 h weekly group session for 8 wks (no mention of home practice) Attrition: 39% 8 wks, 48% 4 mos, 67% 6 mos	Outcomes: B, 8 wks, 4 mos, 6 mos (1) Between-group NS for FIQ, pain, depression (2) Within-group comparisons show significant improvements in all measures, maintained at follow-up (3) First controlled study of qigong for FM (4) Confounded by multiple interventions
(2) Mannerkorpi, Arndow 2004 [18] N = 38; N = 19 qigong, N = 19 normal activities Means: age 45 yrs, FM duration 10 yrs Intervention: qigong (style not reported) + body awareness; 1.5 h weekly group sessions for 3 mos (encouraged to practice at home, but none did) Attrition: 14 (39%)	Outcomes: B, 3 mos (1) Within-group body awareness changes in treatment, but not control group; within-group FIQ improvement in control but not intervention group; all other measures NS (2) Confounded by multiple interventions
(3) Haak, Scott (2008) [19] N = 57; N = 29 qigong, N = 28 wait-list Means: age 53.3 yrs, FM duration 15.4 yrs Intervention: qigong (He Hua), 11.5 h instruction/practice over 7 wks (encouraged to practice 2 × 20 min/day; 2 external qigong sessions) Attrition: 1 (2%) Note: At end of wait-list, subjects also received qigong training; allows for individual and combination group analysis	Outcomes: B, 8 wks, 4 mos (1) Between-group pain, sleep, psychological function all significantly improved at 8 wks, 4 mos (2) Many effect sizes medium-to-large (3) Good reproducibility between immediate and delayed intervention groups (4) Combination qigong group data (N = 56) shows benefits in all domains following intervention; these are maintained at follow-up
(4) Liu et al., 2012 [20] N = 14; N = 8 qigong, N = 6 sham exercise Means: age 56.6 yrs, FM duration 9.4 yrs Intervention: qigong (Liu Zi Jue, 6 Healing Sounds), 2 training sessions, 1 hr weekly group sessions, daily home practice (2 × 15–20 min) for 6 wks; sham had movement but no meditation or sound Attrition: 2/8 in intervention group Note: (1) Only study to use sham exercise. (2) Participants recorded practice time, with moderate-to-high compliance (75–85%)	Outcomes: B, 6 wks (1) Significant within-group effects for pain, fatigue, sleep, FIQ in qigong but not in sham group (2) Between-group effect sizes calculated in the report were large (1–2) in all domains (3) Trial is limited by small numbers
(5) Lynch et al., 2012 [21] N = 100; N = 53 qigong, N = 47 wait-list Means: age 52 yrs, FM duration 9.6 yrs Intervention: qigong (Chaoyi Fanhuan), 3 half-day training sessions, 45 min daily home practice over 8 weeks (home practice reported) Attrition: 12% at 6 mos; 29% at 12 mos Note: (1) At end of wait-list, subjects also received qigong training; allows for individual and combination group analysis. (2) Largest qigong study in FM to date. (3) Study design allowed for two cohorts to be analysed. (4) Numbers allowed for analysis of practice-response relationship	Outcomes: B, 8wks, 4 mos, 6 mos (1) Significant between-group effects for pain, sleep, FIQ, physical and mental function (2) Similar beneficial effects in all domains in the immediate and delayed intervention groups (3) Combination qigong group (N = 73) showed many medium-large effect sizes [15] (4) N = 38 (52%) per protocol (≥5 h/wk); effect sizes in this group uniformly high (0.95–1.67) [15] (5) Comparison of N = 38 with those who practiced minimally (≤3 h/wk) showed significant differences in outcomes in all domains
(6) Maddali Bongi et al., 2012 [22] N = 30; N = 15 qigong, N = 15 Rességuier method Means: age 57.3 yrs, FM duration 7.2 yrs Intervention: qigong style not clear; 2 × 45–60 min sessions wks 1–3, one session for wks 4–7; 7 wks; daily home exercise for 30 min during intervention Attrition: 0% when training commenced, but 8/38 withdrew following randomization Note: (1) Cross-over trial; after first 7 weeks, 1 wk break, then 7 weeks of other method. (2) Comparative trial between two mind–body practices. (3) Initial 7 weeks is comparison trial; remainder considered an add-on trial	Outcomes: B, 7 wks, 15 wks, 6 mos (1) Significant improvements in pain, sleep, FIQ, and mental function in both groups (within-group comparisons) over initial 7 wks (2) No additional improvements at the end of 15 wks following addition of the other method (3) Effects in all domains maintained at 6 mos (4) Effect sizes generally range from 0.7 to 1.5 in both groups [15]

Note: Trials summarized in table used 1990 FM criteria. Abbreviations: B, baseline; FIQ, fibromyalgia impact questionnaire; h, hours; min, minutes; mos, months; NS, non-significant (*p* > 0.05); wks, weeks; yrs, years.

**Table 3:** Summary of other studies of qigong for fibromyalgia.

Study Characteristics	Outcomes, Features
(1) Creamer et al., 2000 [25] Pilot study, open label; N = 28 Means: age 47.9 yrs, FM duration not reported Intervention: 8 × 2.5 h weekly sessions involving education, cognitive/behavioural components (30 min) relaxation/meditation (60 min), qigong (60 min, form not specified), for 8 wks Attrition: 8/28 (29%) did not complete 5/8 sessions	B, 8 wks, 4 mos, 6 mos (1) Significant improvements in FIQ, sleep, pain, and other forms of function after program; benefits generally maintained at 4 and 6 mos (2) Intent-to-treat analysis of results Limitations: high attrition; multiple techniques used as intervention, so not possible to ascribe effects to any particular one
(2) Chen et al., 2006 [23] Pilot study, open label; N = 13 Means: age 49.8 yrs, FM duration 6.2 years Intervention: external qigong applied for 5–7 × 45 min sessions over 3 weeks; monthly maintenance session during follow-ups to 3 mos Attrition: 3/13 (23%) dropped out after 1–3 sessions, data for N = 10 analysed	B, 3 wks, 1 mo, 3 mo (1) Within-group significant improvements in FIQ, pain, depression, anxiety, but not sleep, following qigong (2) Benefits generally maintained at 1 and 3 mos, although there was some rebound (3) Within-subject effect sizes 0.7–1.9 [15] (4) Two cases had such dramatic and persistent benefit following treatment that they considered themselves cured; individual outcomes indicate minimal residual symptomology Limitations: low numbers; dropouts; unclear justification for protocol used; unclear mechanism of external qigong
(3) Lynch et al., 2009 [26] Pilot study, open label; N = 23 Means: age 51.5 yrs, FM duration 12.0 yrs Intervention: Two 4 h training sessions (level 1 CFQ), weekly 1.5 h practice/review sessions; 9 wks (daily home practice 45 min recommended, but not monitored) Attrition: 21 (91%) completed 4 wks, 14 (61%) completed 9 wks, 13 (52%) completed follow-up	B, 9 wks, 3 mos, 6 mos (1) Within-group significant improvements in pain, FIQ and physical function following intervention, and at follow up (effect sizes 0.6–0.9) [15] (2) Analysis conducted on N = 12 (52%) who completed the trial Limitations: dropouts; unclear justification for protocol used
(4) Sawynok et al., 2013 [27] Extension trial to RCT [21], N = 20 Intervention: Level 2 CFQ instruction over 2 × 4 h, and 45 min daily practice (levels 1/2) for a further 6 mos Attrition: 7/20 (35%) Note: (1) Total qigong practice time is at least 12 months, but not necessarily consecutive; 5/13 who completed the extension had continued on with community-based practice following the RCT. (2) Both quantitative and qualitative outcomes, as well as practice times, recorded	Quantitative: For N = 13 who completed extension, significant within-group improvements in pain, FIQ, sleep and function; 5/13 had voluntarily continued with community practice following the RCT and reported practicing 10–15 h/wk Qualitative: Comments recapitulated benefits in qualitative domains; also noted benefits in food allergies, chemical sensitivities, asthma, migraines, blood pressure, vision; several medications discontinued Limitations: low numbers; dropouts; retrospective selection
(5) Sawynok et al., 2013 [28] Case reports, N = 2 females (45, 57 yrs) with FM (10, 20 yrs) who undertook community-based qigong (level 1, level 2 CFQ) at workshops and practiced ≥1 h/day for 6 mos; practice levels up to 1.5–3 h/day following repeat workshops; 3 yrs qigong experience Note: (1) Both had previously tried many other therapies, both conventional and complementary, but remained symptomatic. (2) Both undertook continued qigong training and practice because of health experiences with initial practice (over months)	Case 1: initial improvements in pain, tension, anxiety, food sensitivities, blood pressure; 12 mos: medications and supplements discontinued; 3 yrs: minimal pain (occasional, local), headaches gone, cognition, sleep, fatigue, mood, skin and circulation all improved Case 2: initial improvements in energy and bowel and bladder function; 6–12 mos: vast improvement in pain and other symptoms (including vision); 3 yrs: resumed eating foods previously allergic to, stopped amitriptyline and supplements Limitations: unknown generalizability; retrospective selection
(6) Sawynok and Lynch 2014 [29] Retrospective analysis of qualitative comments by N = 73 who completed initial 6 mo RCT [21] Note: (1) Given that benefits were shown to be related to amount of practice [21], narrative comments for extension trial completers were considered separately in a post hoc manner. (2) Comments for those who practiced per protocol (≥5 h/wk; N = 38), minimally (≤3 h/wk; N = 13), or in between considered as blocks	Narrative: There was a difference in initial experiences (over 6 mos) with qigong by those who completed the extension trial (N = 13) vs. those who did not complete (N = 7); comments recapitulate quantitative domain measures, but also cover other areas Thematic: There was a clear difference in comments on pain, sleep and quality of life by those who practiced per protocol compared to those who practiced minimally; the intermediate group also made positive comments, but these were more moderate in tone Limitations: variable depth of qualitative comments offered by different participants
(7) Sawynok 2016 [30] Case reports (37, 57 and 57 yrs old) of females with fibromyalgia/chronic pain (also other symptoms) of 12–20 yrs duration who undertook extensive qigong practice over 8–15 yrs, practicing 1–3 h/day at times Note: (1) All three experienced improvements in their vision, and the selection of cases was based on this. (2) It would be impossible to conduct a prospective trial involving these amounts of practice over these intervals	Case 1: Commenced qigong in 2008; had FM with multiple issues, all of which resolved over time; currently takes no medications; improvements in vision occured gradually over time (acuity changes 1.5–1.75) Case 2: Commenced qigong in 2006; had FM, arthritis, back pain, sleep apnea, high cholesterol, allergies/food intolerances, irritable bowel syndrome, frequent bouts of pneumonia/infections. Practiced qigong extensively; health improvements in pain and other areas occurred gradually and with different time courses. Currently pain free with improvement in all health areas. Vision changes reflect genetics and surgery. Case 3: Commenced qigong in 2000; multiple health conditions (pain, headaches, sleep disturbance, irritable bowels, food allergies); all health areas improved. Visual acuity improved 2.0 units over first decade of practice in one eye (blind in other eye since birth). Limitations: unknown generalizability; retrospective selection

Note: Trials summarized in this table generally used 1990 FM criteria. However, the onset of FM in several case reports predates 1990, and the condition was a clinical diagnosis rather than a research diagnosis. Abbreviations: B: baseline; CFQ: Chaoyi Fanhuan Qigong; FIQ: fibromyalgia impact questionnaire; h: hours; mos: months; wks: weeks; yrs: years.
